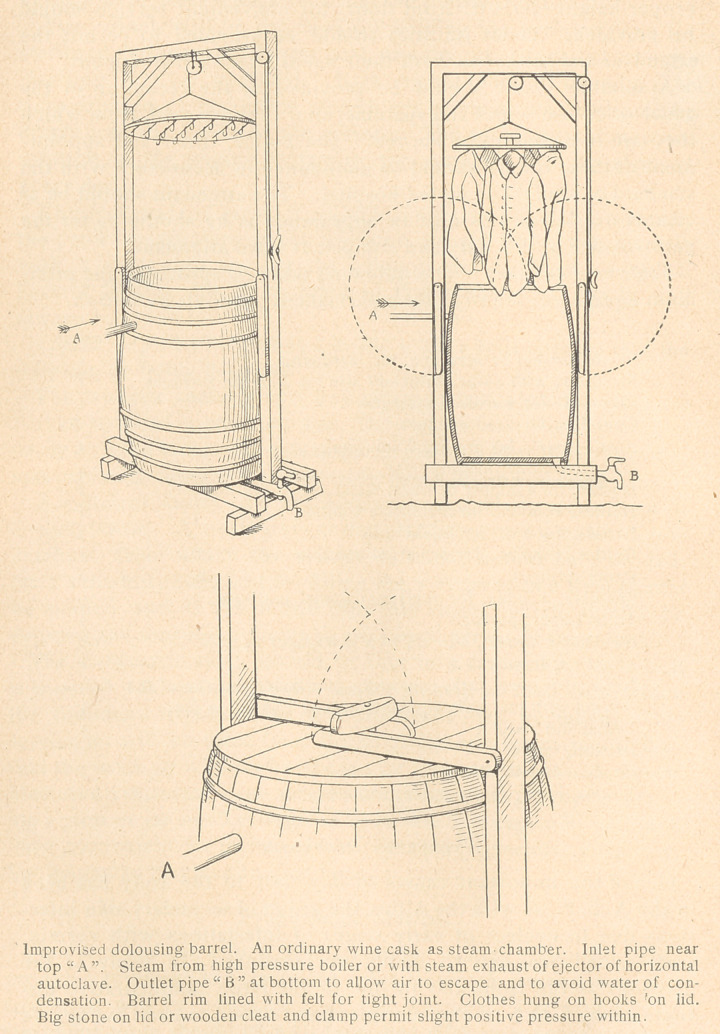# Circulars, Bulletins, and Reports from the C. S. O.

**Published:** 1918-09

**Authors:** 


### CIRCULARS, BULLETINS AND REPORTS

ISSUED FROM THE OFFICE OF THE CHIEF SURGEON OF THE AMERICAN EXPEDITIONARY FORCES IN FRANCE.

*Under this heading will be published extracts from circulars and bulletins issued by the Chief Surgeon of the Medical Department of the American Expeditionary Forces in France. It is believed that these will be of general interest and value to medical officers.*

### EXTRACTS FROM C. S. O. CIRCULARS

### THE NEW PLAN FOR PROMOTIONS IN THE MEDICAL RESERVE CORPS.

1. The Medical Reserve Corps has not heretofore received promotions to which the Corps is entitled by law, because of the many difficulties which have presented themselves in working out a system which would be just and satisfactory.

2. Great inequalities occurred in the original commissioning of Medical Reserve officers by which men of mature age and high standing in the medical profession were made junior to others who were younger and of less professional experience. Further inequalities have been created by the promotion in the United States of younger officers who afterwards came to France with the increased rank which had been denied to members of the Medical Reserve Corps of the A. E. F.

3. A plan has now been prepared in this office which has met the approval of the Commander-in-Chief and which it is desired should immediately be put into operation. This plan recognizes that several factors should be considered in determining the rank of a member of the medical profession coming into the Army in time of war to give voluntary service.

(*a*) The first is age and the length of his professionnal experience, which constitutes, generally speaking, the asset of greatest value to the government which he brings into the service.

(*b*) The second is the length of his active service, which determines his miliiary experience.

(*c*) The third is the character of his military service, and whether it has been distinguished by unusual self-denial, gallantry, efficiency, or hardships which would entitle the candidate to advancement beyond others of the same professional and military experience. On the other hand, this factor may be one of inefficiency or ill conduct which would in justice demand the withholding of promotion, or even separation from the service.

4. In order to accumulate the data for the determination of these factors in each case; it will be necessary to have commanding officers and Senior Medical Officers furnish recommendations in the case of officers of the Medical Reserve Corps serving under them. Individual reports have been requested in the case of each officer serving in France, whether he is considered deserving of promotion or not, except in the case of those under draft age. Officers under the draft age will not be promoted except in special cases where the officer has rendered unusually distinguished service and has been more than a year on active duty.

In making the promotions, the character of the service of the officer will be given consideration as follows :

A. Has it been of a satisfactory and creditable character such as, when his age, professional experience and length of service are considered, would entitle him to a higher grade; or

B. Has it been fairly satisfactory in positions not of great responsability, but not such as would warrant promotion to a higher grade; or

C. Is the officer, on account of professional ignorance, indolence, bad habits, or moral delinquency of any sort, undesirable for the military service ? In this case, as full a statement as is practicable should be made of all the facts throwing light upon the shortcomings of the officer, and it should be stated whether he has been brought before a board of officers under G. O. 45, G. H. Q., 1918.

Policy Governing Promotions. An extract from the letter received by the Chief Surgeon from the Adjudant General of the A. E. F., which has recently been sent out in a circular, explains clearly the recently approved plan for promotion of the Medical Reserve Corps Officers serving with the A. E. F. It has also been extended to the Dental Reserve Corps and the Surgeon General has been requested to adopt it for these Corps in the United States. The corrective promotions authorized in the first paragraph will be made as rapidly as the reports called for are received and then promotions will be made according to the roster. Precedence in the roster will be determined by age and length of service, except that a value will also be given for distinguished service including wounds and decorations received and mention for conspicious gallantry.

The following will be considered the policy that will govern in regard to the promotion of officers of the Medical Reserve Corps in the A. E. F. :

Policy governing promotion of Medical Reserve Officers.

(*a*) All officers of the Medical Corps in Europe will be placed on a roster according to age in each grade. An officer's age will be determined by his actual age plus four months for each month of service.

(*b*) All Lieutenants whose actual age is above thirty-one and who have completed one year's service, shall be eligible for recommendation for promotion to Captain.

(*c*) Promotion in general will be according to seniority, as determined by these rosters.

(*d*) Taking the number of first Lieutenants of the Medical Reserve Corps in the A. E. F. at any time as a basis, the number of officers in grades of Captain and Major shall not be greater than that authorized by the proportion of one Lieutenant to three and nine tenths Captains to one and seven tenths Majors (approximately the proportion between the same grades in the Regular Medical Corps at the time of the passage of the Medical Reserve Law).

(*e*) Recommendation on the part of the military superior of each officer, with a statement that his services have been satisfactory, will be required in each case of recommendation for promotion.

The policy with regard to promotion of officers in the Dental Reserve Corps shall be the same as that outlined above for the officers of the Medical Reserve Corps. The Chief Surgeon is authorized to forward at once any recommendations for promotion which he believes should be made, for the purpose of rectifying inequalities in grade due to mistakes in original appointments.

Form for Report as to the Character of Services and Qualifications of Medical Reserve Officers. 1. Full name and rank. 2. Date of birth. 3. Medical school from which graduated, with date of graduation. 4. Date when ordered into active service on Reserve Corps commission. 5. Previous active military service, either in U. S. Army or with National Guard in U. S. service. 6. Character of service of officer :

A. Has it been of a satisfactory and creditable character such as, when his age, professionnal experience and length of service are considered, would entitle him to a higher grade ; or

B. Has it been fairly satisfactory in positions not of great responsibility, but not such as would warrant promotion to a higher grade ; or

C. Is the officer, on account of professional ignorance, indolence, bad habits, or moral delinquency of any sort, undesirable for the military service? In this case, as full a statement as is practicable should be made of all the facts throwing light upon the shortcomings of the officer, in order that he may be brought before a board for the determination of his fitness for the service. Any available evidence in the form of correspondence or documents which is available should be forwarded in such cases.

(State at beginning of answer whether service has been of Class A, B, or C, and write remarks thereafter.

RECOMMENDATIONS FOR PROMOTIONS. The attention of commanding officers of hospitals and other senior medical officers is invited to the fact that the form on the back of Circular 36 should not be used for the recommendation of Majors, H. R. C., because such promotions take these officers out of the Medical Reserve Corps and into the National Army. Promotions of this sort must necessarily be limited to a small class of specially capable officers, occupying positions of unusual administrative or professional importance. Such recommendations should, when made, be in the form of a special report giving with great fulness all the reasons for the promotion. They should not be made at the request of the officer interested, or except when such promotions are obviously in the interest of the service. The blank form with Circular 36 should be used, therefore, only for Captains and for Lieutenants about the age of 31 who are *Class "A" men.*

The responsibility rests with officers making recommendations to see to it that elderly men who have no administrative capacity, and no unusual professional accomplisments which would fit them for the grade of Major — in other words, men who belong to Class " B ", are not recommended for promotion as Class " A " men. Lieutenants within the draft age should only be recommended for promotion in unusual and exceptional circumstances, where the individual has received a military decoration, or wound, or is a man of very unusual professionnal ability and occupying a position of such importance as to make his promotion of obvious advantage to the service.

PROMOTION AND DEMOTION OF ENLISTED MEN, MEDICAL DEPARTMENT. The commanding officers of Hospital Centers are authorized to promote and demote enlisted men of the Medical Department between the grades of Private and Sergeant First Class, inclusive. They will sign warrants " For the Chief Surgeon ", for men promoted under this authority. The number of men promoted will not exceed the percentages authorized by law. Recommendations for promotions of soldiers of the Medical Department to the grade of Master Hospital Sergeant and Hospital Sergeant will be forwarded to this office for approval.

DIVISION OF LABORATORIES AND INFECTIOUS DISEASES.

1. Circular No. 2, Office Chief Surgeon, A. E. F., dated G. H. Q., A. E. F., November 1917, is amended in so far as it relates to the Director of Laboratories, American E. F.

2. A Division of the Office of the Chief Surgeon, A. E. F., is hereby created, to be known as the Division of Laboratories and Infectious Diseases. This division will be an integral part of the Office of the Chief Surgeon, A. E. F., and will be responsible to him through the Chief of the Division of Sanitation. The general organization of this Division will consist of a Director and the necessary number of assistants. The office of this Division will be located in the city in which the Central Medical Department Laboratory, A. E. F., has been established. Col. J. F. Siler, M. C., N. A., is designated as the Director of the division and the following named officers are designated as his assistants:

Col. George B. Foster, Jr., M. C., N. A., Asst. to Director, Section of Laboratories.

Major R. P. Strong, M. R. C., Asst. to Director, Section of Infectious Diseases.

Major Wm. J. Elser, M. R. C., Asst. to Director, Section of Laboratories.

Major Hans Zinsser, M. R. C., Asst. to Director, Section of Infectious Diseases.

Major P. A. Schaffer, S. G., Asst. to Director, Section of Food and Nutrition.

Major Louis B. Wilson, M. R. C., Asst. to Director, Section of Laboratories.

Capt. Ward. J. Mac Neal, M. R. C., Asst. to Director, Section of Laboratories.

This Division is charged with the following general duties:

*Section of Laboratories:*

*a*) Representative of Chief Surgeon in all matters relating to the laboratory service.

*b*) Organization and general supervision of all laboratories and the assignment of special personnel.

*c*) Advisor to the Supply Division, Chief Surgeon's Office, in the purchase or distribution of laboratory equipment and supplies.

*d*) Publication of circulars relating to standardization of technical methods for protection of specimens and other matters of technical interest to the laboratory service.

*e*) Collection and distribution of literature relating to practical and definite appliances in laboratory methods.

*f*) Collection and compilation of statistics on routine and special technical work done in laboratories.

*g*) Instruction of Medical Department personnel in general and special laboratory technique.

*h*) Distribution and replenishment of transportable laboratory equipment.

*i*) Co-operation and co-ordination with the Chemical Warfare Service, American E. F., and the supply of personnel and equipment.

*j*) Supervision of the collection of museum specimens and photographic records for Medical Department activities.

*Section of Infectious Diseases* :

(*a*) Advisor of the Chief Surgeon in matters relating to the prevention and control of transmissible diseases.

(*b*) Collection and distribution of literature and preparation of circulars relating to methods of prevention and control of transmissible diseases.

(*c*) General supervision of laboratory research.

(*d*) Advisory supervision of all acitvities looking to the control of transmissible diseases, including direct liaison with Division Surgeon.

(*e*) Assignment of specially trained personnel and equipment for the investigation of epidemics or threatened epidemics.

(*f*) Experimental investigation of suggested prophylactic methods for the prevention of infectious diseases and recommendations relative to their general adoption.

(*g*) Collection of epidemiological data on infectious diseases.

(*h*) Co-operation and co-ordination with Water Supply Service, American E. F., in the supervision and control of water supplies.

*Section of Food and Nutrition* :

(*a*) Representing the Chief Surgeon in matters affecting the nutrition of the troops.

(*b*) Investigating army food requirements and consumption.

(*c*) Acting in an advisory capacity in the formulation of rations and dietaries of the American E. F.

(*d*) Inspecting food supplies and mess conditions with troops, hospitals and prison camps.

(*e*) Giving instruction in food inspection and handling, mess management and other measures for the maintenance of nutrition and the conservation of food.

4. The laboratories for the American E. F. will be of two general types, Stationary and Transportable. The Stationary Laboratories will include the Central Medical Department Laboratory, Base Laboratories for sections of the J. O. S. and for selected districts where necessary, Army Laboratories where necessary, Base Hospital Laboratories for individual Base Hospitals, Base Laboratories for Base Hospital centers and Laboratories for Camp Hospitals.

Transportable Laboratories will be organized for Evacuation and Mobile Hospitals and for Divisions. Their equipment will consist of standardized expandable units in chests and their personnel will be specially trained for the duties which they will perform.

5. Instructions concerning the laboratory service, of general interest to all Medical Department units functioning with the American E. F., will be issued in circulars from this office.

6. The Director of the Division of Laboratories and Infectious Diseases is authorized to issue special letters and circulars of instruction governing the organization and activities of this division.

### Prophylactic Administration of Anti-Tetanic Serum.

Circular No. 6 is amended to read as follows:

1. The attention of Medical Officers, A. E. F., is directed to the absolute necessity for the prophylactic administration of anti-tetanic serum (A. T. S.) under the following conditions:

(*a*) Immediately after the receipt of a wound of whatever nature or severity.

(*b*) Upon the recognition of so-called Trench Foot with or without skin abrasions.

(*c*) In cases of frost bite.

(*d*) During operations performed under conditions of unsatisfactory asepsis, e. g., emergency operations, operations for hemorrhoids, fistulae or any conditions where fecal contamination is a possibility.

(*e*) During secondary operations necessary in the course of the treatment of wounds received seven or more days previously.

(*f*) Following the manipulations incident to the reduction of compound fractures or dislocations, after the removal of adherent drains, or any other procedure resulting in a serious disturbance of the healing processes in a wound seven or more days old.

2. One prophylactic dose of 1000 units of Tetanus antitoxin will be given to all wounded whatever, the nature or severity of the wound, as promptly as possible after the infliction of the wound; if a battle casualty, preferably at the Battalion Aid Station. This dose should be given sub-cutaneously, preferably over the lower abdomen. A second dose of 1000 units will be given in every case after an interval of seven days.

3. In severe injuries where prolonged suppurative processes persist, especially when fecal contamination of the wound per rectum or through intestinal fistulae is present and when much tissue necrosis occurs, three or even four doses may be indicated. The attending Medical Officer must bear this in mind and exercise judgment accordingly, in the individual case.

4. There is no objection to the use of 1500 units for the initial and the second prophylactic doses, but doses of 1000 units each afford sufficient protection. (Note. Tetanus Antitoxin from the United States usually contains 1500 units to the dose.)

5. The serum should be administered by, or under the immediate supervision of, a medical officer. If for any reason this is impossible, it should be given by some responsible member of the Medical Department.

6. All injections with amounts and dates, signed by the officer administering them, will be entered on patient's Field Medical Card, by the letters A. T. S., followed by the date and hour. In the case of the freshly wounded the letter T should be marked plainly upon the patient's forehead with an indelible pencil.

7. Absence of any records on the patient's card or face as indicated in the preceding paragraph, is to be accepted as evidence that the A. T. S. has not been given. The first medical officer to assume subsequent control of a patient thus neglected should administer the serum immediately.

8. Medical officers who are thus compelled to administer A. T. S., because of the failure of any medical officer or officers previously responsible for this administration to carry out the above instructions, must make an immediate report of such emissions to the Chief Surgeon, A. E. F., through the Director of General Surgery, with sufficient data to establish the time and circumstances of the omission.

### Anthrax.

The following letter from the Surgeon General, of July 6, 1918, is quoted for your information:

" I am directed by the Surgeon General to inform you that the number of cases of anthrax being reported to this office is sufficient to attract attention at this time. Anthrax, so far as reported, has without exception appeared on the face or neck, and shaving brushes have fallen under suspicion, and in some cases anthrax organism have been isolated from them. For this reason, it is necessary that each case of anthrax coming to your attention be examined critically; that the man's shaving brush, talcum powder and other shaving accessories be obtained; that the organism be sought for with great thoroughness. For the purpose of testing brushes, it is recommended that inoculation of bristles from the brush be made into rabbits, guinea pigs and rats; nothing short of this may give conclusive results. Report should be made to this office of each case, giving the clinical history, the etiology, and the results of the examination of supposedly infected material. The shaving brush or other article from which the anthrax bacillus may be isolated must also be forwarded to this office with full information as to its source, name of the maker, and other data to facilitate its identification.

### Administration of Messes. Function of Dietitian.

The reports of medical inspectors and officers of the Food and Nutrition Section show that the administration of the messes is, as a rule, the least efficient and satisfactory part of hospital administration. The defects noted are a monotonus and ill-balanced dietary, poor service, and lack of cleanliness in the kitchen and the kitchen personnel. These inspections show that Commanding Officers have not made proper use of the agency which is especially intended to correct these defects, that is, — to make proper use of the dietitians who have been assigned to the base hospitals, to use their expert knowledge for the correction of these defects, and to exercise the constant vigilance and attention to detail which is necessary for successful mess administration.

Dietitians are trained experts in nutrition and food preparation. If not trained nurses, they are civilian employees having a status analogous to that of a trained nurse. The function of the dietitian is to supervise the preparation not only of the special diets, but to make out the bills of fare and supervise the preparation of all food furnished by the government. The dietitian has expert knowledge of which the Commanding Officer should make the fullest use for the benefit of his command. She should be able to relieve the mess officer from the burden of details required to secure a well balanced ration, proper variety and preparation, and a good service, The mess officer should make a daily inspection, accompanied by the dietitian and the mess sergeant, to see that the details of good service are carried out fully and completely.

Like all other women of the personnel of a base hospital, the dietitian is under the disciplinary authority of the Chief Nurse.

### Instructions for the use of the Lyster Water Sterilizing Bag.

(*a*) The following instructions for the use of the Water Sterilizing Bag (Lyster) are published for the information of all concerned:

(1) Clean the inside of the bag thoroughly.

(2) Fill it to the white band, with water available.

(3) Place a tube of hypochlorite in an ordnance cup and break the tube with the butt of an ordnance knife. Mix the powder into a smooth paste with a little cold water, using the blade of the knife to break up the lumps. (Hypochlorite tends to lump when added to water and, therefore, special care must be taken to obtain a smooth paste.) Fill the ordnance cup about half full of cold water, stir and pour the nearly clear solution into the water in the bag, keeping the glass in the cup. Stir the treated water thoroughly.

(4) Fasten the cover on the bag and allow the water to stand 30 minutes before use.

(5) Never refill a partially emptied bag. Always empty the water from the bag before filling with fresh water.

(6) Use one tube of powder for every bag full of water. Tubes of hypochlorite are to be obtained from the quartermaster.

(7) Report any difficulties to the Medical Officer.

(8) Keep a record of the treatment attached to the card.

(*b*) Cards containing these directions on waterproof paper are in course of printing and will soon be available for issue.

### Prompt Evacuation of Class "D" Patients.

Attention is directed to the policy of this office with respect to the disposition of all Class "D" patients. It is not intended to hold patients for prolonged periods of observation and study who are clearly destined to fall within this class, no matter how much professional interest they excite.

Such cases should be placed before disability boards promptly for classification, and as soon as they are able to travel by ordinary train they should be sent to Base Hospital No. 8, with a view to their transfer to the United States. If not able to bear travel upon ordinary trains, all such patients should be sent on a hospital train which will be routed regularly to collect such cases as are able to make the journey to the United States.

Therefore, as soon as a patient is classified as of Class " D " he should be considered as destined for transfer to the United States, since the intention is to evacuate to the United States all mutilated and disabled men for treatment, reconstruction, re-education, and final disposition. The necessity for this policy lies in the fact that the hospitalization program in the A. E. F. is based upon a definite priority schedule of building and of housing material, and also of tonnage space for medical supplies on ships from home ports, in direct ratio to the number of troops in France. The hospitalization program in the United States also contemplates the reception of a constant stream of evacuables from the Zone of Operations.

### Biological Products.

The following biological products have been selected by the chief Veterinarian as all that are necessary for the American E. F. Supply Depots and Base Laboratories will carry these only in stock:

(*a*) Serum Antitetanic.
(*b*) Serum Antistreptococcic.
(*c*) Mallein Intradermal.

### Authority to Authorize Expenditures and Approve Vouchers on Medical Department Funds.

Authority to authorize expenditures and to approve vouchers for purchases properly chargeable against Medical Department Funds, in sums not to exceed $250.00, is granted to the Commanding Officers of all Hospital Centers and to the Chief Surgeons of Armies.

The authority to authorize expenditures and to approve vouchers for purchases properly chargeable against Medical Department Funds, in sums not to exceed $100.00, is hereby granted to Chief Surgeons of Army Corps.

### Hospital Trains.

When the Commanding Officer of a hospital is informed of the arrival of a train of patients for his hospital he will send an experienced medical officer and a sufficient number of enlisted men to unload patients from the train. This work is not to be done by the train personnel except in emergency.

Commanding Officers of base hospitals are authorized to issue expendable medical and surgical supplies to the commanding officers of hospital trains, taking the memorandum receipt of the train as a voucher for property return.

---

### EXTRACTS FROM WEEKLY BULLETIN OF DISEASE

### "Three day fever" — "Spanish flu" — Influenza.

The epidemic of respiratory infection which appeared first in the A.E.F. on April 15th in the vicinity of Bordeaux continues to develop in small out-breaks; during the last one 60 cases were admitted in the first 8 days of August. In the early periods of the epidemic, identification of the infecting organism was found to be difficult, but as the smears and swab specimens from nose and throat were taken earlier in the disease, in fact during the stage of congestion and when the constitutional symptoms were developing, the bacteriologists more and more commonly found the Pfeiffer bacillus in generous numbers. The A. E. F. appears to have suffered less than many of the groups subsequently affected, for the epidemic has appeared in a severe form in England and in Switzerland, causing a considerable proportion of serious pulmonary complications which were strikingly absent among the cases reported in May and June in France.

We may expect a continuation or return of influenza in the autumn and winter. The following simple measures put into effect by some medical officers during the past three months with good results are called to the attention of all medical officers as a basis for action.

(a) Early recognition and segregation of all cases of acute respiratory affections or fever in hospital.

(b) Prompt treatment by rest in bed and evacuation of bowels, and light diet at the beginning of the attack.

(c) Forbidding attendance at large public gatherings, "Movie" shows, concerts and dances during period of prevalence of epidemics of colds, coughs or "grippe".

### Diarrhea.

Intestinal flux has been quite prevalent recently in the A. E. F. Whether we call it cholera morbus, dysentery, diarrhea, enterocolitis or acute intestinal indigestion, we can not blink the fact that the causes of practically every case have been preventable and well within the control of the officers and men of the A. E. F. The ingestion of dirty food and water is the simple and the correct explanation of the extensive epidemics which have caused a large burden of unnecessary suffering and inconvenience to our men in every part of France. The dirt has in 99 o/o of the cases been our own dirt, and the food and water have been of our own providing. Feces have got into the food. All varieties of infecting organisms, familiar to dwell are in temperate zones and plenty of tropical organisms have been identified. Among them the commonest have been Shiga, Flexner, Hass Y., Wheeler, Paratyphoid B. and the Entameba Histolytica.

Do not unload the responsibility for summer diarrhea upon the filthy fly; carriers, i. e., sick men with diarrhea, typhoids, dysenteries, etc., have served food in many kitchens. Officers and men, even in parts of France far from the turmoil and disorganization of the recently captured areas south of the Vesle, constantly drink water from unapproved sources in utter disregard of orders issued for their protection. A diarrhea of only one day followed by 3 days of constipation in a negro private of Engineers was found to be due to the Flexner bacillus. Most of those clinically recovered from what seems a simple dietetic diarrhea continued, as do typhoid convalescents, to spread their infection by hand contact with their fecal discharges. "That France has been well seeded must be acknowledged if one will but count the harvest. It is verily in our *own hands* to prevent a continuance or a recurrence.

### Shock and its Remedy.

Whether shock be due to exposure, extreme physical exertion, insufficient food and water, loss of blood, violent and extensive injury, or a combination of these various causes, aid can be given at once whether the patient be a surgical case awaiting operation or a case of psychoneurosis. The latter exhibit many of the physical phenomena of shock better known as accompaniments of physical injuries, but now that we find 10-14 o/o of casualties falling into the group of psychoneuroses we must extend our application of remedy for shock to such of these as are in urgent need.

The following description expresses the results of careful study and mature judgment of our ablest physiologists and surgeons. The sample directions are worthy of close attention :

*The Warming of Shocked Men.* There is unanimous testimony that loss of body heat is conducive to the production or increase of traumatic shock, and that warmth is an effective means of preventing shock, or lessening it when already established. The sevely wounded man sweats even to a degree that may wet his clothing; he may have a diminished blood pressure from on-coming shock or hemorrhage; and he is liable to exposure during the examination of his wound and in the ambulance. All these conditions lead to lowering of body temperature and thus to lessening of the chances of survival. To preserve or to restore normal body temperature the following precautions should be remembered: (1) In *examining wounds*, cover with a blanket the part finished before starting the examination of another part. (2) Badly wounded men suffering from shock or hemorrhage are usually thirsty. Give them *hot drinks*, preferably hot coffee, or tea, with sugar. Do not give alcohol in any form as its use aggravates and does not relieve symptoms. Avoid giving drinks if there is an abdominal wound with possible penetration of the stomach or intestine. (3) Use *three blankets* if there is danger from cold. To obtain the best service from them fold them in such a way that this provides for four layers of blanket beneath and four layers over the patient. A water-proof sheet around the blankets will ckeck loss of heat. (4) In placing *hot water bottles* keep in mind two considerations: that the rapidity of heat conduction depends on difference of temperature, and that more value is obtained from a hot water bottle by using both sides. For quick heating apply the warmth preferably to the coldest parts, i. e., to hands and feet. If only two hot water bottles are available, put one at the feet, the other on the belly with the hands over it. If three more are available place them between the arm and chest on either side, and between the thighs. In a " push ", when supplies may be insufficient, canteens temporarily taken from a salvage heap may be used as hot water bottles. Be careful to avoid a heat that may burn the skin.

(5) *At the hospital* set on a cot or a bed the stretcher bearing the patient. Between the cot or mattress and the stretcher is a space of about 5 inches. If the patient is cold and shocked, lay a blanket over him and let it fall down at the sides and at the foot of the stretcher; the space is thus enclosed except at the head end. Now apply heat at the foot end. This may be simply done by cutting a square tin box, such as is used for dried potatoes in the A. E. F., by supporting the box so that the upper opening connects with the enclosed space under the stretcher, and by lighting in the box three or more candles, or a small can of solid alcohol (partly covered if the flame is too great). In a stationary hospital with electricity a frame 3 feet long and 18 inches high may be constructed and wired for six electric lamps which project inward from the frame. The frame, set over the patient, is covered with blankets and the current turned on. The cot must be kept level; otherwise the heat will not be evenly distributed. *Do not overheat the patient.* He needs fluid and should not be caused to lose it by unnecessary sweating. When he is warm, carefully remove his clothing and lay him in the warmed bed.

### Some suffered that others may learn: Gas Prevention.

After a recent gas attack the following notes were made for the information of the chief Surgeon:

" The bombardment began at ten P. M. and lasted an hour, during which time shells were discharged at the rate of about 15 per minute. The night was warm and dry with a slight breeze blowing from the north which later changed to the northwest. Following the bombardment there was a lull of nearly three hours during which time occasional shrapnel and artillery shells were exploded. At about one A. M. the gas bombardment was renewed with increased vigor. This attack lasted about fifty minutes during which time gas shells were discharged at a greater rate than during the earlier attack. " Mustard " seems to have been the principal gas used, with a possibility of a slight mixture with other gasses.

The principal cases of the casualties during this attack are as follows:

(1) Delay in applying respirators, and their promiscuous removal during, and premature removal following, one bombardment.

(2) Relying on poorly constructed dugouts.

(3) Failure to awaken sleeping men.

(4) Permitting men to enter an old mill in the gassed area and to remove their masks.

After careful analysis of the conditions associated with this attack, it is the opinion that many of the casualties were the result of individual carelessness and to a certain extent poor Company Gas Discipline. Of course the fact must not be lost sight of that the members of these organizations had been heavily engaged with the enemy for the past two or three weeks, during which time they were subjected to many hardships which resulted in a general lowering of their physical condition, thereby rendering them in a fit state for the actions of poisonous gases. Nevertheless, considering everything in connection with this bombardment, the lowered physical condition of the men, the nature of the attack, darkness, heavy underbrush, poorly constructed dugouts and permitting men to enter a gas saturated building, etc., it is the opinion that with proper care many of these casualties could have been averted.

From interviews with casualties resulting from this attack, few seemed to be familiar with the question of mask removal during the following gas attacks. Some were of the opinion that they removed their masks immediately after the bombardment ceased; others stated they did not remove them until they saw their officers without masks, and still others said they removed them at intervals during the bombardment for the purpose of obtaining better vision or because the respiration was uncomfortable. Few had received orders of any kind from officers or others relative to removal of masks.

The dugouts appear to have been one of the principal causes for the gas casualties. These dugouts from all accounts were but mere holes of different sizes dug in the side of the trenches, some capable of holding two men, others more; all were protected by hanging curtains made of shelter halves dropped in front. The men entered these places thinking they were safe and removed their masks; as a result many became gassed. Many men were gassed in an old mill which was in the midst of the gassed area. Following the bombardment the men entered this place, removed their masks and laid down on the gas-saturated hay-covered floor to rest. All stated the odor of gas was very strong at the time, but inasmuch as their officers were present without masks they thought the place safe. Many men were gassed because they were not awakened when the gas attack began. Others stated they smelt the gas long before any gas alarm was sounded.

Attention is again invited to the subject of long hair in connection with gassed cases. This matter has been taken up before and recommendation made that all men in the front area be compelled to have the hair on their heads closely cropped. I am firmly convinced that long hair on the head has much to do with the harboring of poisonous gases, thereby being responsible for many men being gassed after the removal of their masks. All medical officers interrogated relative to this subject are of the same opinion. It is again recommended that all men serving in the front areas be compelled to have their heads closely cropped.

Yes! it is admitted that Company discipline does not rest with the medical officer, but the line officers will measure the importance of medical necessities by the determination the M. O.'s put into their demands. Demand Prevention, i. e., Gas Mask Discipline.

### Three Lung Irritants. — A Note from the Chemical Warfare Service.

All the gases that act as lung irritants cause essentially the same type of pathological effects. The damage to the alveoli of the lungs which results from their action is followed by the rapid onset of acute pulmonary œdema, and it is the accumulation of fluid in the lungs which constitutes the immediate danger to life, since it interferes with the respiratory change in the lungs and leads to severe want of oxygen, which is indicated either by deep cyanosis or by pallor and collapse. The deeply cyanosed or leaden colored facies, the quickened respiration, rapid pulses, restlessness and profuse expectoration make a clinical picture which is characteristic in the majority of severe cases.

*Phosgene* is one of the most intense lung irritants known. Though acute pulmonary œdema may develop with great rapidity after exposure to a very strong dose of phosgene, it is of the utmost importance to recognize that in the case of less massive doses a very striking delay, even amounting to hours, frequently elapses between exposure to the gas and the onset of severe symptoms, and that during this interval the case may complain only of slight discomfort and may appear to be in good condition. This delayed action characterizes many of the lung irritant gases.

*Chlorine* : A much stronger concentration of this gas is needed to cause severe pulmonary œdema, or even lachrymation, than is the case with phosgene. It is far more irritant to the respiratory passages than phosgene.

*Dichlor-Ethyle-Sulphide* (*Mustard-Gas*) : Differs entirely from the above mentioned lung irritants. Phosgene kills directly and speedily by flooding the lungs with œdema fluid. Mustard gas kills at a later period, and death, when it does occur, is an indirect result of secondary lung infection, except in a few examples where the patient is asphyxiated by obstruction with bronchial sloughs.

### Venereal prophylaxis.

What might be mildly dignified as a " System of Venereal Prophylaxis " has yielded such satisfying results in practice that it seems to be based on correct principles. The point of departure for both attendant and patient is placed in the teaching that " Venereal prophylaxis, being a major operation like catheterization, is entitled to professional consideration ". The attendant, therefore, must be placed in such a relation with the patient that the latter will see no humor in his position as a " Professor of Prophylaxis ".

This end is easily attained by establishing the Prophylaxis Station in a respectable, clean and well appointed location and providing such apparatus, in addition to the technical equipment, as will furnish the means of conducting the operation with at least as much dignity and formality as is practiced in a barber shop. Such an adjunct is provided in the form of a "Prophylaxis Stool" which requires the patient to assume the sitting posture, brings him

Prophylaxis Stool. In use at Camp Hospital No 19. Giving good service.

under the control of the attendant and permits a definitely outlined procedure which both the attendant and the patient may recognize and respect as an operation. A drawing of the prophylactic stool which has met these ends is presented herewith. The object of the device is two-fold.

(*a*) To provide a definite and simple means of bringing the patient and attendant into formal relation during the procedure of the treatment.

(*b*) To provide a convenient position for the patient which will permit a thorough application of the different steps of treatment without soiling the clothes or surroundings. The box compartment is intended to hold the material and equipment when not in use and to place them in a convenient position for operation.

It has been found by practice that this stool makes it easy for the attendant not only to perform with integrity the several steps of his operation, but to grow so confident in his art and so interested in its practice that he becomes an efficient and enthusiastic instructor of interested pupils. The technic prescribed for the use of this stool is as follows: (From a circular issued by Commanding Officer, Camp Hospital 19.)

I. *The Duty of the Attendant.*

A Medical Department Attendant will be on duty at the prophylaxis station at all times. He will be definitely instructed in all details of the technic of prophylaxis by the Surgeon, who will inspect the pratice of the attendant often enough to assure himself the duties are properly performed. The prophylaxis treatments will be given by the attendant in accordance with the following directions. A patient will not be permitted to administer the treatment to himself. Before beginning the treatment, a prophylaxis card will be carefully prepared with the name of the patient and other entries. The signature of the patient will be written on the top of the back of the card. The identification tag of the patient will be inspected to verify his name and organization, if the patient be not known to the attendant. The Medical Officer in charge will inspect and sign the cards daily, to insure that all required data are recorded. If, for any reason, the attendant is unable to administer a treatment in the proper manner, in accordance with the prescribed method, he will write on the prophylaxis card the reasons why treatment could not be properly given.

II. *Preparation of the Patient.*

The patient will be required to urinate in the presence of the attendant immediately before the treatment is commenced. He will be placed in a sitting position with his trousers lowered below the knees, and a basin placed below and between his thighs so as to catch all fluids and to protect his clothing and the surroundings. A pint of warm water will be placed in the basin and the patient will receive a wipe of gauze with which to wash thoroughly his genitals with soap and water. The attendant will then examine the patient for discharge from the urethra and sores on the penis. If either of these conditions be discovered, a record of the observation will be made on the prophylaxis card. The attendant will drop slowly on the penis, from a bottle, about a tablespoonful of liquid soap, while the patient thoroughly washes the penis with the wipe and the clean water in the pan. While washing is continued the attendant will see that the foreskin is retracted and that all parts about the head and body of the penis, together with the scrotum, are thoroughly washed. After the washing with soap and water is finished, the attendant will pour slowly on the penis from a bottle at least one half pint of a warm solution of 1 : 1000 bichloride of mercury, during which time the patient will thoroughly wash all parts of the penis in the same manner as directed above in using soap and water. The gauze wipe used in the washing will be placed in a proper receptacle to be disposed of later by a prescribed recovery process or by burning. The penis will be allowed to dry by evaporation while the injection is being given, as it is not advisable to wipe off the bichloride solution.

### III. *Injection.*

The injection will always be given by the attendant. A few drachms of a 2 % protargol solution will be poured from the bottle into a small glass from which the syringe will be charged. One drachm of this protargol solution will be drawn into the syringe. The attendant will hold the meatus between the thumb and finger of the left hand, pressing from top to bottom, and place the nozzle of the syringe in the meatus with the right hand. The solution will be slowly injected until the syringe is empty, while the attendant continues to hold the penis. The patient will then take hold of his penis and relieve the attendant of any further part in the injection. The patient will hold his penis, so as to retain the solution, for five minutes by the clock, being careful to press only on the extreme end so as to allow the fluid to come into contact with all parts of the urethra. The meatus should be held only tightly enough to retain the fluid while allowing an occasional drop to escape. This precaution of not holding the meatus too tightly is very important because the location of the infection is liable to be in the very end of the urethra, and compressing the urethra too tightly prevents the solution from coming into contact with the very surfaces where infection is most likely to occur.

After the time limit of five minutes has expired, the pressure on the meatus is released and the protargol solution is allowed to escape without pressure on the urethra to expel the last drop which it is desired should remain.

IV. *Inunction.*

If the penis is not perfectly dry after the washings with bichloride solution, the patient will receive a newspaper towel with which to dry it thoroughly. The attendant will remove from the small container, by means of a small wooden spatula about two inches long, one-half drachm of a 33 % calomel ointment which he will smear over the surface of the penis while the patient retracts the foreskin and turns the penis so that the ointment can be applied to all parts. After the ointment is transferred from the spatula to the penis, the spatula will be placed in a proper retainer to be burned. A new spatula will be used for each case and the old one will always be destroyed. The attendant will make these spatulae from suitable pieces of wood.

After the ointment is thus applied to all parts of the penis by the attendant, the patient will carefully and thoroughly rub the ointment into all parts of the skin covering the penis, being very careful to withdraw the foreskin and to make the rubbing very thorough about the meatus, head and neck of the penis. This rubbing should be continued until the ointment has disappeared, leaving only a colorless greasy film on the skin of the penis. While the ointment is being rubbed into the forepart of the penis, a small portion, about the size of a pea, will be pushed into the meatus and left there. Any excess of the ointment which remains will be rubbed towards the base of the penis and the scrotum. After the inunction is completed and remains as only a greasy film on the skin, the foreskin will be drawn down over the head of the penis.

The attendant will then provide a piece of toilet paper, of newspaper, with which the patient will wrap the penis to prevent the ointment from being rubbed off and to protect his clothing. The patient will be directed not to urinate within a period of 4 or 5 hours after the treatment. Liquid soap, water and a newspaper towel will be provided by the attendant to permit the patient to wash his hands, by which operation the treatment is completed.

V. *Materials.*

The *syringes* will be sterilized by boiling whenever practicable; otherwise each syringe must remain in a solution of 1 to 1 000 bichloride of mercury for at least one hour after it has been used on one patient, before it may be used on another. One dozen syringes should be furnished to provide a capacity of one dozen prophylaxis treatments in one hour, when sterilization is effected by the antiseptic solution. When the syringes are sterilized by boiling, 10 minutes in boiling water is required for preparing a syringe to be used again after it has once been used.

Two vessels will be provided for holding the syringes, one for sterilized syringes ready to be used, and the other for used syringes awaiting to be sterilized. Each vessel should contain 1 t 1 000 solution of bichloride of mercury, some of which should be drawn into the barrel of the syringe. The bichloride of mercury solution in the vessels containing the syringes must be changed every day.

The *bichloride of mercury solution*, (1 : 1 000) used for washing the penis, preparatory to the injection, should be kept in a bottle with a split cork so that the solution can be freely dropped. Several bottles should be filled so that a sufficient supply will always be at hand.

*Liquid soap* will be kept in a bottle with a split cork so that the soap can be freely dropped.

The wooden *spatulae* will be kept in a clean closed vessel or a corked bottle of appropriate size. They may be sterilized by boiling or by 1 : 1000 bichloride solution. A Medical Department enamel spit cup with cover makes a suitable vessel for this purpose.

A sufficient supply of a 2 % *protargol solution* should be kept in a stock bottle. A small glass or dish, holding not more than a few drachms, from which the syringe is filled, should be provided. Only a quantity sufficient for filling the syringe at one time should be poured out of the bottle into this vessel, which should be emptied and thoroughly cleaned after each period of treatment.

The *ointment* in stock should be kept in a covered jar in which it is issued. A small, covered, service jar will be used, from which the ointment required for each treatment will be taken. This service jar will be filled from the stock jar as often as required so that the stock jar will not be opened for each treatment. A regular Medical Department issue white enamel covered spit cup, containing a glass or jar small enough to fit in this spit cup, will be found most convenient for this service. (Details of Quarters, Furniture, Equipment to be given in next issue)

*Prophylactic Station.*

*Quarters and Furniture*: A comfortable waiting room should be provided adjoining the treatment room in which patients awaiting treatment will be protected from the weather. The prophylaxis treatment room should be well furnished with an appropriate table upon which the treatment materials are placed, and a cupboard or suitable shelving for storage of stock, in keeping with the standard of a well appointed surgical dressing room. A urinal placed at proper height (2-1/2 ft. from floor) for convenient service should be provided. Toilet facilities should be provided to permit the attendant and patient to wash their hands after each treatment.

9. A suitable vessel should be provided for the reception of the gauze or cotton wipes and spatulae, after they are used, from which they are removed to be burned. The police, order and appointment of these rooms should be maintained in accordance with the standard of a well conducted hospital. The attendant should be as cleanly dressed and as neat in appearance as he should be for the performance of this duty as a competent ward attendant.

*Table of Equiment* : The following table recapitulates the minimum equipment required for a permanent prophylaxis station.

| | Number |
|---|---|
| Stove, alcohol or kerosene, for sterilizing | 1 |
| Sterilizer, with cover, sufficient size for 1 dozen syringes | 1 |
| Container for sterilized syringes | 1 |
| Container for used syringes | 1 |
| Small vessels for protargol solution | 2 |
| Basins, enamel, medium size | 4 |
| Bucket, for a supply of warm water | 1 |
| Bottles, 1000 cc. for bichloride solution | 1 |
| Bottles, 1000 cc. for liquid soap | 2 |
| Bottles, 1000 cc. for protargol solution | 1 |
| Penis syringes | 12 |
| Spatulae, wooden, for applying calomel ointment | 50 |
| Toilet paper, package | 1 |
| Newspaper towels, size 1/4 of a newspaper page | 100 |
| Toilet paper rack | 1 |
| Newspaper towel rack, consisting of a board the size of the towel with two nails after the form of a Shannon file | 1 |
| Urinal and stand | 1 |
| Dressing table | 1 |
| Cupboard, with shelves | 1 |
| Towels, linen, for attendant and cleaning | 12 |

### Pyodermia Decreasing.

One of the important causes of *days lost* to the army has been in the A. E. F., as with the Allies, the group of secondary skin infections due to neglect of early stages of infestations with vermin, scabies, and lice, and to bodily uncleanliness. In many of the divisions at the front and in one of the large administrative districts of the S. O. S. the pyodermias have ceased to exist owing, we are informed by the dermatologists who especially delight in the refinements of nomenclature, to the following simple procedure. At venereal inspection twice each month all men strip to the waist and drop breaches to knees. A glance at axillae, elbows and

Improvised dolousing barrel. An ordinary wine cask as steam-chamber. Inlet pipe near top "A". Steam from high pressure boiler or with steam exhaust of ejector of horizontal autoclave. Outlet pipe "B" at bottom to allow air to escape and to avoid water of condensation. Barrel rim lined with felt for tight joint. Clothes hung on hooks on lid. Big stone on lid or wooden cleat and clamp permit slight positive pressure within.

buttocks supplements the examination of the penis. An interested sergeant can be trusted with the skin inspection while the surgeon hunts for venereal lesions. A dermatologist should attend these inspections whenever practicable. In one month pyodermias ceased to exist in one large administrative district. The bath is another important place to catch the elusive parasites, and unless bathing is organized and adequate the men have not had the 88 o/o chance to escape infestation to which they are entitled. Here is a case of good bathing where inspection is part of the job : Enlisted strength of Battalion 1952, baths given (in one week) 2488, "C. C.'s", cootie cases, found 26, scabies cases found 5.

*Le Gérant :* O. Porée. Paris — Imp. Lahure, 9, rue de Fleurus.

## Figures and Tables

**Figure f1:**
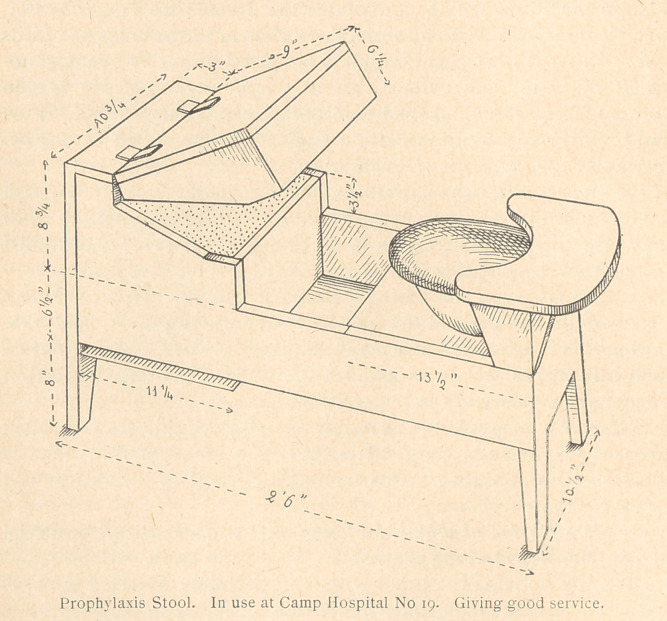


**Figure f2:**